# Removal of Heavy Metal Ions from Aqueous Solution Using Biotransformed Lignite

**DOI:** 10.3390/molecules28135031

**Published:** 2023-06-27

**Authors:** Jianguo Cheng, Shanfei Zhang, Chen Fang, Litong Ma, Jianguo Duan, Xu Fang, Rihong Li

**Affiliations:** 1 Inner Mongolia Engineering Research Center of Comprehensive Utilization of Bio-Coal Chemical Industry, Baotou 014010, China; 2 School of Chemistry and Chemical Engineering, Inner Mongolia University of Science and Technology, Baotou 014010, China; 3 Shandong Shengli Bioengineering Co., Ltd., Jining 272000, China; 4 Inner Mongolia Tongwei High Pure Crystal Silicon Co., Ltd., Baotou 014010, China

**Keywords:** lignite, heavy metal ion adsorbents, biotransformation, kinetics, thermodynamics

## Abstract

Heavy metal pollution caused by industrial wastewater such as mining and metallurgical wastewater is a major global concern. Therefore, this study used modified lignite as a low-cost adsorbent for heavy metal ions. Pingzhuang lignite was dissolved and modified using *Fusarium lignite* B3 to prepare a biotransformed-lignite adsorbent (BLA). The O, H, and N contents of the BLA increased after transformation, and the specific surface area increased from 1.81 to 5.66 m^2^·g^−1^. Various adsorption properties were investigated using an aqueous solution of Cu(Ⅱ). The kinetic and isothermal data were well-fitted by pseudo-second-order and Langmuir models, respectively. The Langmuir model showed that the theoretical Cu(II) adsorption capacity was 71.47 mg·g^−1^. Moreover, large particles and a neutral pH were favorable for the adsorption of heavy metal ions. The adsorption capacities of raw lignite and BLA were compared for various ions. Microbial transformation greatly improved the adsorption capacity, and the BLA had good adsorption and passivation effects with Cu(II), Mn(II), Cd(II), and Hg(II). Investigation of the structural properties showed that the porosity and specific surface area increased after biotransformation, and there were more active groups such as –COOH, Ar–OH, and R–OH, which were involved in the adsorption performance.

## 1. Introduction

Industrialization is accompanied by activities such as refining, mining, smelting, and agriculture that can contaminate the surrounding soil and water with heavy metals [1,2]. In China, there are approximately 12,000 mine tailings that store wastewater and residue from industrial processes, and they can easily pollute the surrounding environment [3,4,5]. Moreover, unlike organic pollutants, heavy metals are not degraded by microorganisms, and they accumulate step by step in organisms along the food chain [6]. Therefore, it is necessary to remove low concentrations of heavy metals from wastewater before it is discharged to satisfy discharge standards [7]. If wastewater is insufficiently treated or discharged without treatment, then it poses a significant threat to our living environment and health.

Conventional heavy metal wastewater treatment technologies include ion exchange, reverse osmosis, adsorption, chemical precipitation, membrane filtration, and electrodialysis [8,9,10]. However, these methods have disadvantages such as high processing costs and technical complexity, and there is a risk of secondary pollution. Therefore, new methods have been developed for the treatment of wastewater polluted by heavy metals. Among these methods, nano-iron doped lignite is considered to be a simple, effective, and economic approach, hence, this technology has attracted considerable research attention, after being doped, the adsorption capacity of lignite to cadmium increased by 36% [11].

Lignite is the youngest type of coal, and the degree of coalification is between that of peat and bituminous coal. It is brownish-black in appearance and has strong reactivity [12]. Moreover, it contains many active groups, such as carboxyl (–COOH), hydroxyl (–OH), phenolic hydroxyl (Ar–OH), aldehyde group (–CHO), and carbonyl (–C=O) groups [7,13,14,15], has a large cation exchange capacity, large specific surface area, and high chemical and biological stability. Thus, it can effectively reduce the migration and bioavailability of heavy metals in soil and industrial wastewater [16].

In recent years, various studies have investigated the mechanisms by which lignite adsorbs metal ions, and a variety of theories have been proposed including complex chelation, covalent adsorption, ion-exchange, surface adsorption, and chemisorption [9,17,18]. In particular, chelation and ion exchange are the most widely accepted. The adsorption capacity of raw lignite for heavy metal ions is weaker than that of synthetic ion-exchange materials, which greatly limits its application in water treatment and soil remediation. However, the use of lignite for the adsorption of heavy metal ions remains of interest owing to its low cost [19,20,21]. In recent years, various treatment technologies have been used to modify lignite to improve its porosity, active functional group content, and stability [22,23,24]. Thus, modified lignite has proven to be valuable in pollution control processes such as the removal of heavy metal ions and organic pollutants. Doskoil et al., studied the adsorption and removal of Pb^2+^, Cu^2+^, Cd^2+^, and Zn^2+^ ions from an aqueous solution by natural lignite. They found that the adsorption capacity of Pb is the largest, reaching 30 mg/g, and the adsorption capacity for Cu is 26 mg/g [8]. Zhang et al., reported on bentonite and lignite can passivate heavy metal ions in soil, after treatment; the content of lead in corn root decreased by 3.4%–33.7% [25]. Beksissa et al., found that lignite treated with acid has a better adsorption effect on Cr(VI) [26].

Existing literature shows that the adsorption capacity of raw lignite is very low. Therefore, this work aims to improve the ability of lignite to adsorb heavy metals via microbial depolymerization. This is achieved by converting low-cost Pingzhuang lignite from Inner Mongolia into a lignite-based adsorbent with greater adsorption capacity, higher porosity, and more active groups. Then the modified lignite is used to adsorb and remove a mixture of heavy metal ions such as Cu(II), Mn(II), Cd(II), and Hg(II) from an aqueous solution. This shows that the treated lignite is an excellent adsorbent for heavy metal ions.

## 2. Results and Discussion

### 2.1. Evaluation of Proximate Analysis Result of Lignite Samples

Table 1 shows that the chemical composition of the BLA was significantly different from that of raw lignite. After microbial treatment, the oxygen, hydrogen, and nitrogen contents increased from 20.43%, 4.87%, and 1.46% to 25.74%, 6.23%, and 1.97%, respectively. The oxygen, hydrogen, and nitrogen contents of the BLA are important factors in its ability to adsorb heavy metal ions. By contrast, after microbial treatment, the carbon and sulfur contents decreased. Moreover, the volatile matter and moisture contents decreased significantly, which improved the stability of the adsorbent [26]. The Brunauer–Emmett–Teller (BET) surface area (m^2^·g^−1^) increased by a factor of more than three after treatment, from 1.81 to 5.66 m^2·^g^−1^, and a higher BET surface area is associated with a greater number of active groups that can react with heavy metal ions.

### 2.2. Adsorption Kinetics of BLA with Cu(II)

#### 2.2.1. Adsorption Kinetics

**Figure 1:**
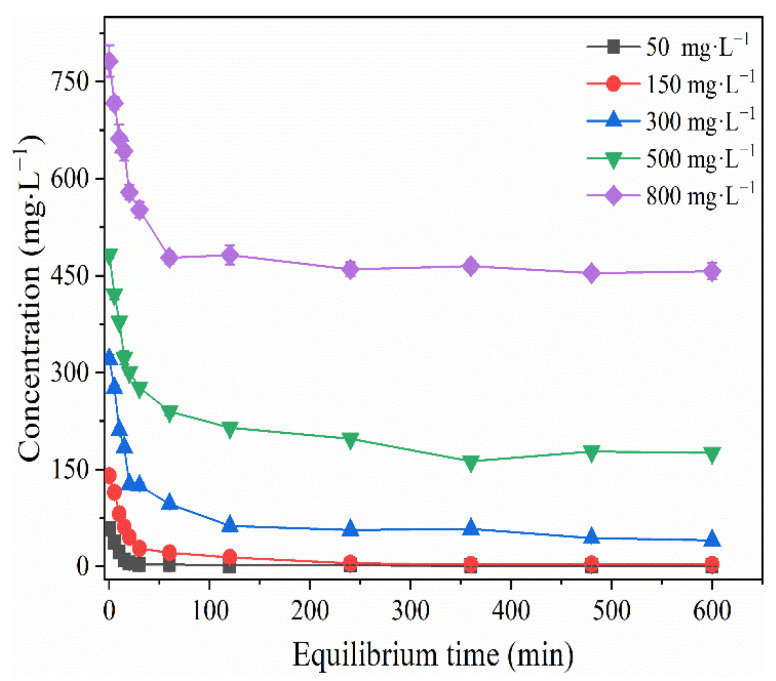
Adsorption effect of BLA for Cu(II) with different initial concentrations. (The experiments were conducted with BLA = 0.5 g, pH 6.0 ± 0.2, and T = 24 ± 1 °C.).

**Figure 2:**
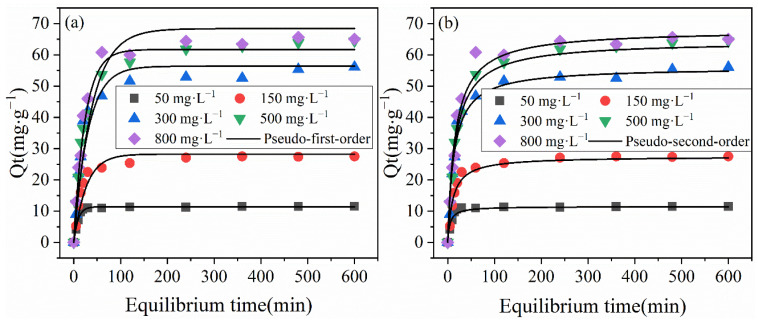
Kinetics of Cu(II) removal by the BLA. (Fitting with (**a**) the first-order reaction kinetic model and (**b**) the pseudo-second-order reaction-rate model).

To explore the adsorption properties of BLA for heavy metal ions, the removal capacity was investigated for initial Cu(II) concentrations between 50 and 800 mg·L^−1^ at pH 6.0 and T = 24 ± 1 °C. The results are shown in Figure 1. Then, the first-order reaction kinetic and pseudo-second-order reaction-rate models were used to simulate the adsorption process. The dynamic simulation results are shown in Figure 2a,b.

When the initial Cu(II) concentration was 50 mg·L^−1^, the adsorption of Cu(II) by the BLA was complete and reached equilibrium within 120 min. Moreover, the removal rate was 97.1%. When the initial Cu(II) concentration was 150 mg·L^−1^, it took 240 min to reach equilibrium. At initial Cu(II) concentrations of 150, 300, 500, and 800 mg/L, the removal rates after 240 min were 96.1%, 82.3%, 59.0%, and 41.2%, respectively. When the adsorption dose of lignite was constant, the removal rate decreased as the Cu(II) concentration increased. The Cu(II) adsorption rate in the initial 30 min was fast, owing to the availability of active centers on the BLA that quickly captured the heavy metal ions. The maximum Cu(II) adsorption capacity at equilibrium was approximately 65.0 mg·g^−1^. The Cu(II) adsorption capacity of the BLA was much higher than that of raw lignite. The maximum adsorption capacity was three times greater than that reported by Jellali et al. for Cd(II) and Cu(II) using raw lignite from the Cap Bon region (northeastern Tunisia) [27]. Moreover, it was approximately 16 times greater than that reported by Milicevic for Cu(II) using low-cost adsorbent Kolubara lignite [21]. However, it was lower than the value reported by Havelcova for Cu(II) using raw lignite [17].

The adsorption kinetics were investigated to understand the relationship between the rate at which heavy metal ions were captured and the adsorption capacity of the adsorbent. Furthermore, the microporous diffusion coefficient and adsorption process were analyzed using fitting kinetics [28,29]. In this study, pseudo-first-order and pseudo-second-order kinetic modeling were used to estimate the rate-determining step during the adsorption of Cu(II) ions by the BLA. Figure 2 shows the evolution of the adsorption capacity over time, and the corresponding fitting-rate constants for each model are listed in Table 2.

The results show that the pseudo-second-order model was most consistent with the experimental data. The *R*^2^ values of the adsorption capacity curves for all the concentrations were between 0.976 and 0.992. The equilibrium adsorption capacities *q_e_*_2_ of the pseudo-second-order model were 11.5, 27.5, 56.0, 64.4, and 66.3 mg·g^−1^, which were much closer to the experimental results of 11.48, 27.5, 56.1, 64.5, and 65.0 mg·g^−1^, respectively, than those obtained from the other model. Thus, the BLA removed heavy metal ions via chemisorption, and the absorption capacity rate depended on diffusion in the adsorbent particles [26,30].

#### 2.2.2. Adsorption Isotherms

To study the thermodynamic adsorption characteristics of the BLA, adsorption isotherm studies were conducted for Cu(II) with ion concentrations ranging from 20 to 1000 mg·L^−1^. The other conditions were the same as those used to investigate the adsorption kinetics. For these tests, the contact adsorption equilibrium time was set to 240 min because it was sufficient for the heavy metal ions to reach equilibrium in the kinetics experiments.

**Figure 3:**
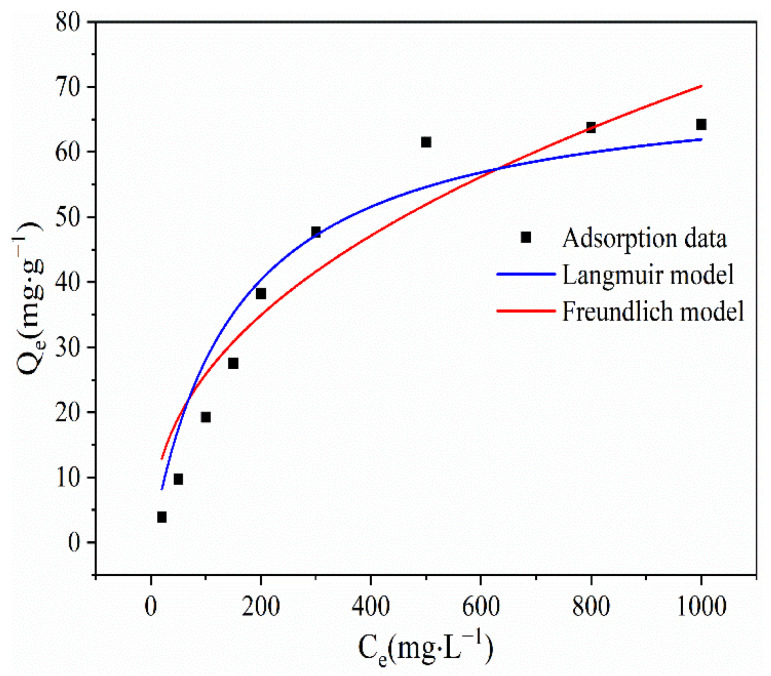
Adsorption isotherms for the Langmuir and Freundlich adsorption models. (The fitted models are shown in comparison to the experimental data).

The Langmuir and Freundlich isotherm models were used to fit the experimental data of Cu(II) adsorption by the BLA. The results are shown in Figure 3, and the fitting parameters and fitness of the models are given in Table 3. The determination coefficient *R^2^* of the Langmuir model was 0.95, which was much higher than that of the Freundlich model, which was 0.91. The *R_L_* values of the Langmuir model were between 0.10 and 0.88 (0 < *R_L_* < 1). This indicated that monolayer adsorption occurred through chemical bonding, and the adsorption of Cu(II) on the BLA was positive [24,31,32]. Fitted by the Langmuir model, the maximum equilibrium adsorption capacity *Q_m_* of Cu(II) was 71.47 mg/g, but the experimental result was 65.0 mg·g^−1^. The *Q_m_* deviation between the experimental result and the Langmuir model was 9.9%. In addition, the Freundlich constant 1/*n* for Cu(II) was 0.433, which is between 0 and 1, and the *K* value was 3.25, which put it in the range of 1–10. This indicates that the adsorption of molecules by the BLA was a favorable process [32].

### 2.3. Factors Affecting Adsorption

#### 2.3.1. Effect of Particle Size Distribution on Adsorption

**Figure 4:**
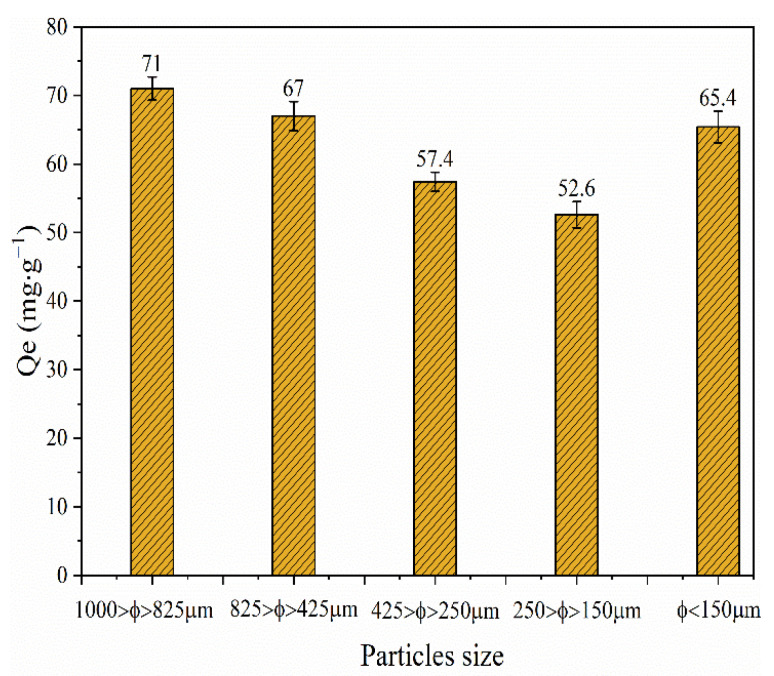
Effect of BLA particle size on Cu(II) removal efficiency. The experiments were conducted with *C*_0_ = 500 mg/L, BLA = 0.5 g, *t* = 240 min, pH 6, and *T* = 24 ± 2 °C.

It is well known that adsorption is a surface phenomenon of solid adsorbents to surrounding molecules and ions and the particle size distribution of the adsorbent has a substantial effect on the adsorption properties. The effect of the size fraction on the Cu(II) adsorption was investigated under the following experimental conditions: initial Cu(II) concentration 500 mg·L^–1^, pH 6.0, 0.5 g of BLA, 240 min contact adsorption time, and room temperature (24 ± 1 °C). Figure 4 shows the relationship between the amount of Cu(II) adsorbed by the BLA and the particle size distribution. At the same time, BET adsorption specific surface area, adsorption pore volume, and pore size of BLA with different particle sizes are shown in Table 4. As the particle size decreased, the Cu(II) adsorption capacity first decreased and then increased. The greatest adsorption (71.0 mg·g^−1^) was observed at the greatest granulometry (1000 > Φ > 825 μm). When the particle size distribution was between 150 and 250 μm, the adsorption capacity was lowest (52.6 mg·g^−1^). This phenomenon can be explained by the BET adsorption specific surface area, adsorption pore volume, and pore size of BLA displayed in Table 4, With the decrease in particle size, the specific surface area, adsorption pore volume, and pore size all tend to decrease, Therefore, For the adsorbents dominated by mesoporous adsorption, the adsorption capacity decreases with the decrease in specific surface area.

**Figure 5:**
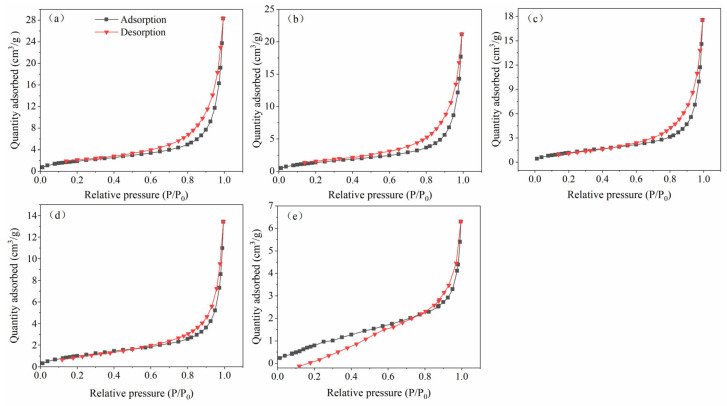
The adsorption isotherms of BLA with different particle sizes (**a**): 1000 > Φ > 825 μm; (**b**): 825 > Φ > 425 μm; (**c**): 425 > Φ > 250 μm; (**d**): 225 > Φ > 150 μm; (**e**): 150 μm > Φ.

Furthermore, when the particle size was less than 150 μm, the adsorption capacity was 65.4 mg·g^−1^. The adsorption capacity suddenly increases, which may be related to the change in adsorption mode. The BET low-temperature N_2_ adsorption isotherms of BLA with different particle sizes are shown in Figure 5. The results showed that the hysteresis isotherm of the BLA sample has no obvious saturated adsorption platform, indicating that the pore structure is very irregular, mainly medium pores and large pores, and contains fewer micropores. With the decrease in the particle size of lignite, it is easier to be dissolved by microorganisms, the pore structure formed by excessive dissolution is less, and the adsorption of metal ions changes from mesoporous adsorption to lamellar adsorption. In addition, this may be because, after microbial dissolution and transformation, the lignite with larger particles had more adsorption pores and a greater pore volume. If the particle size is large, then the pore solution and pore distribution dominate the adsorption process; however, if the particle size is small, then more active sites are exposed and the specific surface area dominates the adsorption process.

#### 2.3.2. Effect of pH

Lignite has a high oxygen content there are many hydroxyl and carboxyl groups in its skeleton structure. These groups are the main active groups involved in the adsorption of metal ions onto lignite-derived adsorbents. Changes in the pH of the system will affect the solubility and state of the heavy metal ions, and the surface charge and other chemical properties of the adsorbents [20,33,34].

Metal cations in aqueous solution form different hydrolysis products, and the cations exist in different forms depending on the solution pH. When the pH is low, copper ions exist as Cu(II), and when the pH is high they exist as Cu(OH)^+^ and Cu(OH)_2_^(s)^. Therefore, the pH of the experimental solution system was kept between 2 and 6 for this part of the study. In addition, the experiment was conducted with an initial Cu(II) concentration of 500 mg·L^−1^, 0.5 g of BLA, particle size of <150 μm, contact time of 240 min, and at room temperature (24 ± 1 °C).

**Figure 6:**
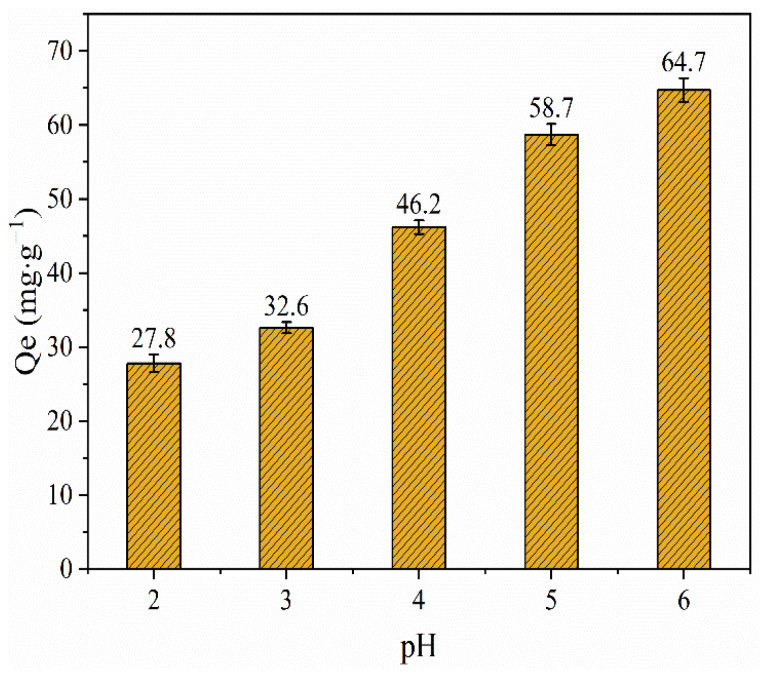
Effect of solution pH on Cu(II) adsorption using BLA. The experiments were conducted with *C*_0_ = 500 mg/L, BLA = 0.5 g; *t* = 240 min, *T* = 24 ± 2 °C, and particle size <150 μm.

The effect of pH on the adsorption efficiency of Cu(II) onto the BLA is shown in Figure 6. With the increase in pH, the adsorption capacity of Cu(II) increased gradually and reached the maximum at pH of 6. This can be explained by the dissociation degree of surface-active groups of the BLA. As the pH value increased, it became easier for Cu(II) to replace the hydrogen ions in the active groups of the BLA, hence, the adsorption capacity increased. Similar results have been reported by other authors [8,17,21].

### 2.4. Adsorption of Four Heavy Metal Ions

**Figure 7:**
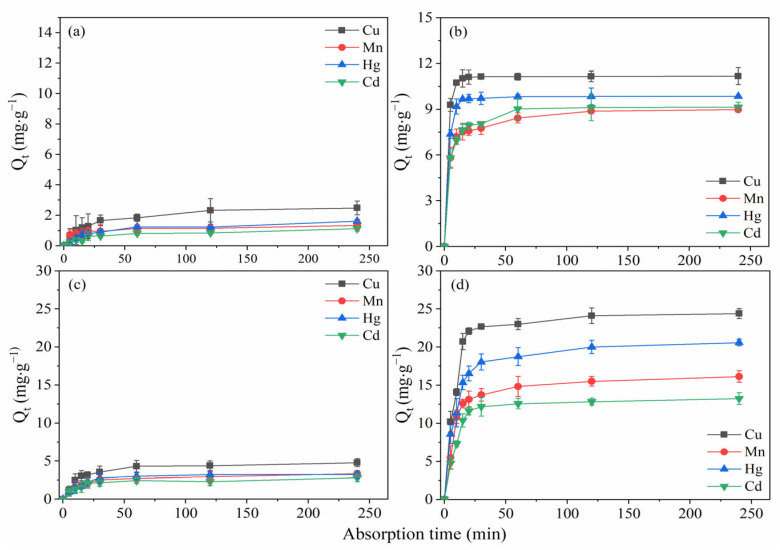
Comparison of heavy metal adsorption characteristics of raw lignite and the BLA. Adsorption with an initial concentration of 50 mg·L^−1^ for (**a**) raw lignite and (**b**) the BLA. Adsorption with an initial concentration of 150 mg·L^−1^ for (**c**) raw lignite and (**d**) the BLA.

It is important to understand the adsorption characteristics of transformed lignite adsorbents with different heavy metal ions. Therefore, mixed ionic solutions of Cu(II), Mn(II), Hg(II), and Cd(II) with different concentrations (50 and 150 mg·L^−1^) were used to investigate the selective adsorption characteristics of raw lignite and the BLA. The solutions were prepared and left to reach equilibrium for 24 h. Then, 100 mL of the mixed ionic liquid was transferred to a 250 mL glass flask for the adsorption test. A contact time of 240 min was selected, the pH was 6.0, and samples were taken at different times to determine the concentration of the residual heavy metal ions in the solution. The experimental results are shown in Figure 7.

Comparing the results showed that the adsorption capacity of the BLA was much higher than that of raw lignite. When the raw lignite was used to adsorb a mixture of heavy metal ions from a solution with an initial concentration of 50 mg·L^−1^, the adsorption capacity of raw lignite to Cu(II), Mn(II), Hg(II), and Cd(II) once the solution reached equilibrium were 2.5, 1.3, 1.6, and 1.1 mg·g^−1^, respectively. In comparison, when the BLA was used the adsorption capacity was 11.2, 9.0, 9.8, and 9.1 mg·g^−1^, respectively. The average removal rate of the four heavy metal ions achieved using the BLA was 93.4%, which was six times higher than that achieved by raw lignite (15.5%).

When the initial concentration of the mixed heavy metal ions was 150 mg·L^−1^, the maximum adsorption capacity was achieved with a contact reaction time of 240 min. The maximum adsorption capacities of the BLA for Cu(II), Mn(II), Hg(II), and Cd(II) in the mixed solution were 24.4, 16.1, 20.6, and 13.2 mg·g^−1^, respectively. However, the adsorption capacity of raw lignite was less than 5 mg·g^−1^. Thus, the heavy metal ion adsorption capacity and removal ability of lignite was greatly improved by microbial transformation. Furthermore, the experimental results showed that the adsorption capacity of the BLA for the four heavy metal ions followed the order Cu(II) > Hg(II) > Mn(II) > Cd(II). This phenomenon may be related to the electron absorption ability and atomic mass of the different metal cations.

### 2.5. Morphology and Composition of the BLA

**Figure 8:**
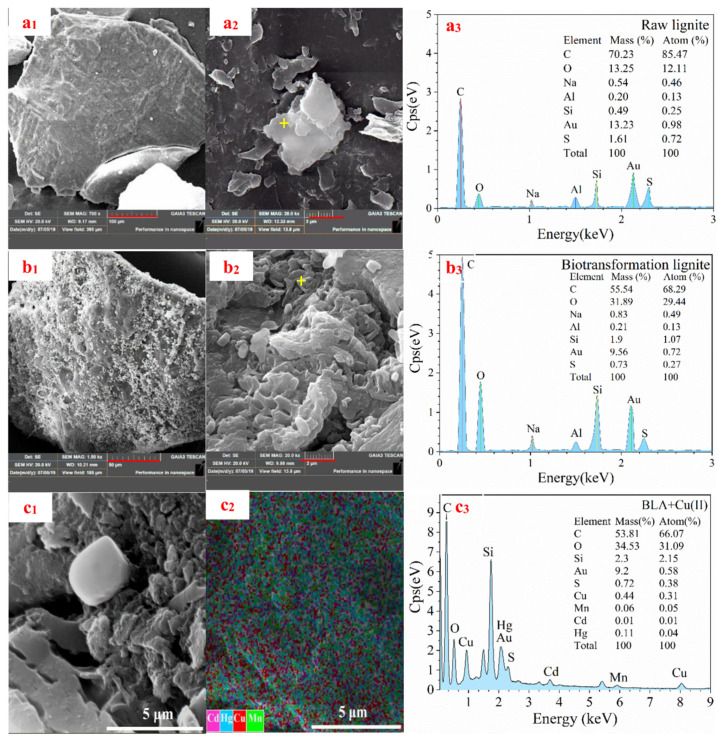
SEM-EDS images of raw lignite and the BLA. (SEM-EDS images of (**a_1_**–**a_3_**) raw lignite, (**b_1_**–**b_3_**) the BLA, (**c_1_**–**c_3_**) SEM-EDS results for BLA adsorbed heavy metal ions).

To explore the mechanisms by which lignite adsorbs heavy metal ions, SEM and energy-dispersive X-ray spectroscopy (EDS) were used to characterize the lignite before and after microbial treatment. The surface morphologies of the lignite samples were observed at 700× and 20,000× magnification. The morphologies of raw lignite and the BLA are shown in Figure 8.

The surface of the raw lignite was smooth with few pores and cracks (Figure 8a_1_,a_2_). In contrast, the surface of the BLA was rough with many dissolved pored and attached microbial cells (Figure 8b_1_,b_2_). This explains the increase in the specific surface area. Thus, it may be concluded that during treatment part of the lignite structure is degraded and dissolved by the microorganisms, which forms pores on the surface, increases the specific surface area, and exposes active adsorption groups. This was confirmed by the EDS results, as shown in Figure 8a_3_,b_3_,c_1_–c_3_. The carbon, oxygen, and sulfur contents of raw lignite were 70.23%, 13.25%, and 1.61%, respectively. After microbial treatment, these values were 55.54%, 31.89%, and 0.73%, respectively. At the same time, the EDS map scanning result showed LAB could adsorb heavy metal ions very well, and the adsorption capacity of Cu(II) was the strongest among the four ions. The results were consistent with the experimental results of heavy metal adsorption.

Thus, the microbial action reduced the carbon and sulfur content substantially and increased the oxygen content. This increase in the degree of oxidation may increase the number of oxygen-containing active groups, such as carboxyl, hydroxy–phenolic, and carbonyl groups, on the surface, thus increasing the heavy metal ion adsorption capacity and ion exchange ability.

### 2.6. FTIR Analysis and Molecular Mechanism of Adsorption Cu(II)

**Figure 9:**
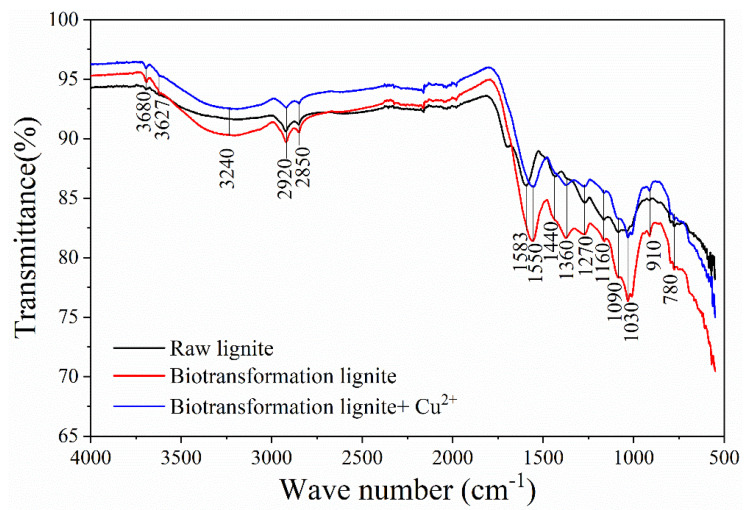
FTIR spectrums of various samples. (FTIR spectrums of raw lignite, the BLA, and the BLA after adsorption of Cu(II).

To investigate the mechanism by which the BLA adsorbed heavy metal ions, FTIR analysis was conducted before and after Cu(II) adsorption. To illustrate the effect of microbial transformation on the adsorption of heavy metal ions, raw lignite was also analyzed. All the FTIR results are shown in Figure 9.

Hydroxyl and carboxyl groups constitute important active centers for adsorption in lignite. Comparing the FTIR spectrums of raw lignite and the BLA shows that the absorption peaks of the active functional groups changed substantially. The sharp peaks at 3680 and 3627 cm^−1^ were associated with phenols or alcohols and O–H stretching vibrations. The wide absorption peak at 3240 cm^−1^ and the sharp peaks at 1583, 1550, 1440, and 1360 cm^−1^ were associated with Ar–OH, carboxylic groups, N–H, O–H, and C=O stretching vibrations [6,35]. There were also absorption peaks at 1270, 1160, 1090, and 1030 cm^−1^ in the lignite, which can be attributed to the presence of C–O, ester of alcohols, carboxylic acid groups, and carboxylic acids [36,37]. The absorption peaks at 1000–650 cm^−1^ were associated with C–H bending vibrations of the ortho- and meta-substituted aromatics [6]. After microbial transformation, the intensity of the absorption peaks associated with carboxylic groups, alcohols, and phenolic hydroxyl groups (Ar–OH and R–OH) increased significantly. Moreover, the absorption peaks of the functional groups moved to lower wavenumbers. Thus, after microbial treatment, more active adsorption sites were exposed and more intermolecular hydrogen bonds were formed, which was favorable for the adsorption of metal ions. Analyses of the FTIR spectra of BLA + Cu(II) revealed sharp peaks between 1600 and 1000 cm^−1^, and the wide absorption peak at 3240 cm^−1^ decreased considerably compared to the peak before adsorption. This may be because the functional groups were involved in the adsorption of heavy metal ions. This indicates that heavy metal ions were chemically adsorbed by the BLA [38,39].

**Figure 10:**
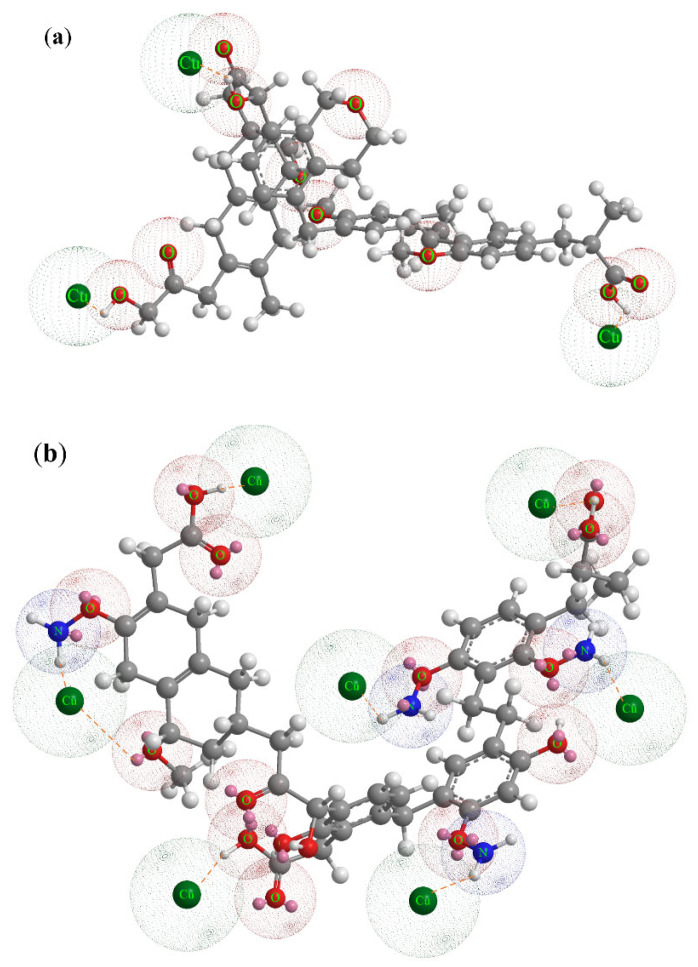
Model of Cu(II) adsorption by the basic structural unit of lignite and BLA. (**a**): Simulation diagram of Cu(II) adsorption by basic structural unit of raw lignite; (**b**): Basic structure model of BLA adsorption simulation diagram of Cu(II)).

The results of FTIR showed that before and after the adsorption of Cu(II) by BLA, the absorption peaks at 1583, 1550, and 1030 cm^−1^ changes obviously. The absorption of these parts was caused by the changes of carboxylic groups, N–H, carboxylic acids, and phenolic hydroxyl in lignite. These groups could react with heavy metal ions such as ion exchange, chelation, and complexation, thus adsorbing and passivating the free metal ions. Taking the basic structural model of lignite constructed by Wender in 1976 as an example (shown in Figure 10a) [40]. In his basic unit model of lignite, there are only three hydroxyl groups and one carboxyl group. Therefore, there are few active sites for the adsorption and passivation of heavy metal ions by raw lignite. Hypothetical after the action of microorganisms, the basic structure of lignite can be broken by biological enzymes to form more active adsorption centers such as amino groups, phenolic hydroxyl groups, carboxyl groups, etc., the basic structure model of BLA is shown in Figure 10b, which can absorb and passivate more metal ions, taking Cu(II) as an example. The adsorption of Cu(II) will lead to the weakening of the molecular vibration of the corresponding active sites or the shift of the absorption peak, which can be proved by FTIR analysis results.

## 3. Materials and Methods

### 3.1. Material, Chemicals, and Equipment

Pingzhuang lignite was mined from the Yuanbaoshan Mining area, Chifeng City, Inner Mongolia, China. The lignite was dried at 80 °C in a vacuum oven for 24 h, crushed, then shaken through a 60-mesh screen to obtain a lignite sample with a grain size of less than 0.5 mm. The lignite sample was packed in a flask and sterilized at 121 °C for 20 min and then left to stand. The chemicals CuSO_4_·5H_2_O, HgCl_2_, MnCl_2_·4H_2_O, and Cd(NO_3_)_4_·4H_2_O were purchased from Merck & Co., Germany. All the solutions were prepared with double distilled water, and different concentrations were prepared according to the adsorption experiments. All other chemicals were of analytical grade and were used as received. Solution pH was adjusted using 1 mol·L^−1^ HCl or NaOH.

**Figure 11:**
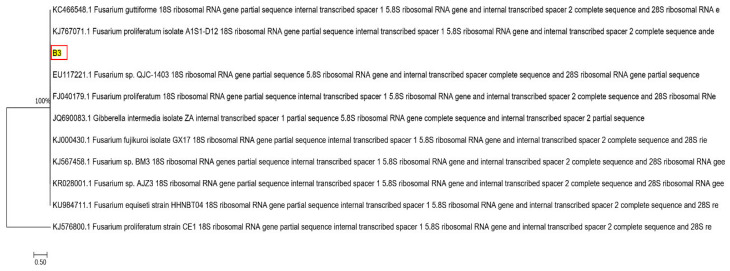
Phylogenic tree of *Fusarium lignite* B3.

The fungus strain used in this study was isolated from Inner Mongolian lignite in our laboratory. The DNA was extracted and amplified with the primers ITS1 (50- TCCGTAGGTGAACCTGCGG-30) and ITS4 (50-TCCTCCGCTTATTGATATGC-30). The polymerase chain reaction (PCR) product was analyzed using the advanced 3730XL analysis system, and the strain was genetically identified by Meiji Biological Co., Ltd. (Shanghai, China). The GenBank^®^ accession number was KJ767072.1, and according to the identification report, it belonged to the species *Fusarium proliferatum* and the strain was *Fusarium lignite* B3 (Figure 11).

Notably, it showed an outstanding ability to degrade lignite. The *Fusarium lignite* B3 was preserved in a glycerol medium at −20 °C in a refrigerator. Before the lignite biosolubilization experiments, it was reactivated and purified in potato dextrose agar (PDA) media (obtained by washing, peeling, and chopping 200 g of potatoes, adding 1 L of deionized water, boiling for 30 min, filtering through gauze, and adding 20 g of glucose and 15 g of agar; the natural pH was used) at 28 °C three times [41,42,43].

The equipment included a pH meter (EL-20, Mettler Toledo, Zurich, Switzerland), elemental analyzer (2400, Perkin Elmer, Waltham, MA, USA), Fourier-transform infrared spectrometer (FTIR; Spectrum 3, Perkin Elmer, Waltham, MA, USA), atomic absorption spectrometer (AAnalyst 800, Perkin Elmer, Waltham, MA, USA), scanning electron microscope (SEM; GAIA3, Tescan, Jebrno, Czech Republic), surface area apparatus (ASAP-2460, Micromeritics, Norcross, GA, USA), high-speed centrifuge (Multifuge X1R, Thermo Fisher, Scientific, Inc., Waltham, MA, USA), ventilated stirring fermentation system (GS8000-5L/A, Guangshi, Shanghai, China), constant temperature oscillator, electronic weighing balance, and vacuum drying oven.

### 3.2. Preparation of Biotransformed-Lignite Adsorbents (BLAs)

**Figure 12:**
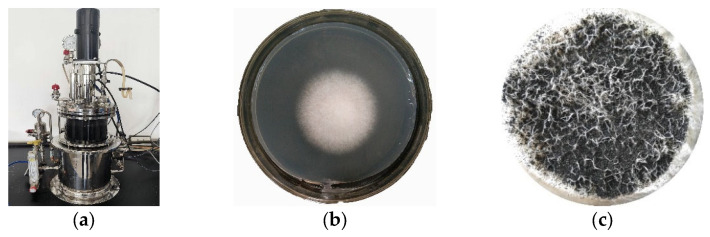
Preparation of BLAs by fermentation system (**a**), colony morphology of *Fusarium lignite* B3 (**b**), and it can grow on the surface of lignite (**c**).

Lignite biomodification was conducted under aerobic conditions using a liquid PDA medium and *Fusarium lignite* B3. To amplify the *Fusarium lignite* B3 culture, it was used to inoculate a 5.0 L ventilated stirring fermenter containing 3.5 L of PDA media without agar. A temperature of 28 °C, sterile-air ventilation flow rate of 150 mL·min^−1^ and a stirring rate of 200 rpm were maintained for 48 h. Then, 300 g of lignite was added to the fermenter under aseptic conditions, and the fermentation state was maintained for an additional 10 days, the experimental results showed that the dissolution conversion rate of lignite by *Fusarium lignite* B3 reached the highest value after 10 days of treatment (Figure 12). The dissolved products and BLA were separated by centrifugation at 4500 rpm for 20 min. The BLA was washed with deionized water three times, then dried at 80 °C. The surface of the dried BLA was characterized to investigate the heavy metal ion adsorption capacity of lignite. It should be noted that dissolved products can be used to separate useful chemicals; however, this is not described in this work.

### 3.3. Adsorption Experiments

All the adsorption experiments of heavy metal ions by lignite were carried out using a batch technique at ambient temperature in an aqueous solution. Three parallel experiments were performed for each adsorption experiment.

The main experimental methods used in this study were as follows. First, 0.5 g of BLA and 100 mL of heavy metal liquid with a known concentration were added to a 250 mL glass flask. Then, the adsorption experiment was conducted in a constant temperature oscillator with an oscillation frequency of 180–200 rpm to keep the lignite particles suspended in solution, and the single adsorption experiment lasted for 600 min. Once the adsorption was complete, the solid–liquid separation was carried out with a 0.45 µm cellulose acetate filter. The residual heavy metal ions in the solution were determined via atomic absorption spectrometry. The adsorptions of different heavy metal ions in solution were recorded at different emission wavelengths using suitable hollow cathode lamps. The characteristic absorption wavelengths were 324.8, 279.5, 253.7, and 228.8 nm for Cu(II), Mn(II), Hg(II), and Cd(II), respectively. The adsorption of heavy metal ions by the lignite samples was measured according to the change in the content of heavy metal ions in the solution before and after equilibrium adsorption [15,17,27]. The amount of adsorption can be expressed by the adsorption capacity equation, it can be expressed as:

(1) $$q_{t}=\frac{\left(C_{0}-C_{t}\right)\times V}{m_{l}}$$

In Equation (1), *q_t_* (mg·g^−1^) is the adsorption amount of heavy metals at any time *t* (min), *C*_0_ (mg/L) is the concentration of heavy metal ions in the initial solution without adsorbent, *C_t_* (mg/L) is the residual concentration of heavy metal ions in the solution at *t* time after the addition of adsorbent, *V* (L) is the volume of solution containing heavy metal ions in the experimental group, and *m_l_* (g) is the amount of lignite adsorbent added to the experimental solution.

The effects of different experimental conditions on the adsorption performance were analyzed. (I) The performance of raw lignite and BLA were compared. (II) The effect of particle size was investigated using BLA ground to <850, 850–425, 425–250, 250–150, and >150 µm. (III) Contact times between 0 and 180 min were tested. (IV) The effect of heavy metal ions concentration was determined by varying the concentration of the initial solution from 50 to 1000 mg·L^−1^. (V) Finally, the effect of the pH of the initial aqueous solution was tested over the range 2.0–6.0 using 0.1 mol·L^−1^ HCl and 0.1 mol·L^−1^ NaOH [6,17,27].

### 3.4. Adsorption Kinetics and Isotherm Model

#### 3.4.1. Determination of the Adsorption Kinetics

To evaluate the adsorption kinetics of BLA for heavy metal ions, the first-order reaction kinetic and pseudo-second-order reaction-rate models were used to fit the adsorption data over time [26,31,44,45]. In recent years, the first-order reaction kinetic model has frequently been used to evaluate the adsorption of pollutants in wastewater. It is given by the equation

(2) $$\frac{dq_{t}}{dt}=k_{1}\left(q_{e}-q_{t}\right),$$

where *q_e_* (mg·g^−1^) is the adsorption amount of heavy metals at equilibrium, *q_t_* (mg·g^−1^) is described in Equation (1), *k*_1_ (min^−1^) is the equilibrium rate constant for the kinetic model.

Integrating Equation (2) with the boundary conditions of *t* = 0 to *t* = *t* and *q_t_* = 0 to *q_t_* = *q_t_*, yields Equation (3) [26]:

(3) $$\log\left(q_{e}-q_{t}\right)=\log q_{e}-\frac{k_{1}}{2.303}t.$$

The pseudo-second-order kinetics can be described as:

(4) $$\frac{dq_{t}}{dt}=k_{2}{\left(q_{e}-q_{t}\right)}^{2}$$

In the pseudo-second-order reaction-rate model, the kinetics process of the adsorption reaction is fitted by the amount of heavy metal ion adsorbate on the surface of the adsorbent at time *t* and equilibrium, and the adsorption rate is proportional to the number of active centers and porosity on the surface of the adsorbent [26,31]. Integrating Equation (4) for the boundary conditions adsorption time t from 0 to t and adsorption amount qt from 0 to qt. Final Equation (4) can be rearranged to obtain:

(5) $$\frac{t}{q_{t}}=\frac{1}{q_{e}}t+\frac{1}{k_{2}q_{e}^{2}}$$

where *q_t_* (mg·g^−1^) is the amount of adsorbate on the adsorbent at any time *t* (min) and *q_e_* (mg·g^−1^) is the equilibrium adsorption capacity. *k*_2_ (g·mg^−1^·min^−1^) is the rate constant of adsorption, which can be determined from the slope and intercept of a plot of *t/q_t_* against *t* in experimental data [31].

#### 3.4.2. Determination of the Adsorption Isotherm

The adsorption isotherm was usually used to study some adsorption characteristics of adsorbents, such as adsorption-specific surface area, pore volume, and pore size distribution. It also provided information about the interaction mechanisms between the biomodified lignite adsorbent and heavy metal ions in the adsorption equilibrium process. In this part, the Langmuir and Freundlich isothermal models were used to describe the experimental results for the adsorption characteristics of Cu(II) by the BLA [26,31,46]. The Langmuir model assumes that the adsorption groups are uniformly distributed on the surface of the adsorbent, that only one layer of solute molecules is adsorbed on the surface of the adsorbent, and that the adsorption is irreversible. Moreover, the maximum adsorption capacity occurs when the monolayer is saturated. In this study, the Langmuir model reflected that the adsorption of monolayer Cu(II) occurred on the homogeneous surface of the BLA sample and that the mixed heavy metal ions did not interact with each other during adsorption [31]. The Langmuir model is described linearly as Equation (6)

(6) $$\frac{C_{e}}{Q_{e}}=\frac{1}{Q_{m}K_{L}}+\frac{C_{e}}{Qm}$$

where *Q_m_* and *K_L_* denote the Langmuir constants, that is, the saturated monolayer adsorption capacity and the adsorption equilibrium constant, respectively. Moreover, *Q_e_* is the amount of Cu(II) adsorbed at equilibrium and *C_e_* is the concentration of Cu(II) adsorption equilibrium solution [26,44].

The characteristics of the Langmuir constants can also be used to express the affinity between the solutes (Cu(II)) and adsorbent (BLA) in terms of a dimensionless separation factor of equilibrium *R_L_*. This is given by

(7) $$R_{L}=\frac{1}{1+K_{L}C_{0}}$$

where *C*_0_ is the initial concentration of solute Cu(II). The *R_L_* criteria are as follows:

Separation factor *R_L—_*Adsorption characteristics of adsorbent Langmuir isotherm;

*R_L_* > 1—Unfavorable adsorption;
*R_L_* = 1—Linear adsorption;
0 < *R_L_* < 1—Favorable adsorption;
*R_L_* = 0—Irreversible adsorption.

The Freundlich isotherm model is an empirical model based on the assumption that adsorption occurs on heterogeneous surfaces and that the active adsorption sites have heterogeneous surface energy [26,47]. Moreover, it assumes that the adsorption capacity is related to the concentration of Cu(II) at equilibrium. The model is expressed linearly as

(8) $$\ln q_{e}=\ln K+\frac{1}{n}\ln C_{e}$$

where *K* is an approximate indicator of the adsorption capacity and 1/*n* is the adsorption intensity.

### 3.5. Characterization Techniques

FTIR spectroscopy was used to assess the functional groups present in raw lignite and BLA before and after adsorption. After drying, the lignite samples and KBr were ground into powders (diameter < 2 µm). The lignite power and KBr were mixed with a ratio of 1:200 (*w*/*w*) and then pressed into sheets using a tableting machine. Finally, the pressed sheets were scanned using light with wavelengths of 4000–550 cm^−1^ and a resolution of 1 cm^−1^. Each sample was scanned 32 times [35,48,49].

The differences in the surface morphology of the BLA before and after microbial modification and the adsorption of heavy metal ions were investigated via SEM/EDS. The samples were washed with anhydrous ethanol and dispersed, fixed on a conductive adhesive surface, naturally air-dried, and then characterized using field-emission SEM/EDS (FE-SEM; Tescan GAIA3, Tescan company, Jebrno, Czech Republic) [50]. The elemental composition of the lignite samples was investigated using an element analyzer (PE PerkinElmer 2400; Perkin-Elmer, Waltham, MA, USA).

## 4. Conclusions

Biotransformed lignite was investigated as a potential adsorption passivator for the removal of heavy metal ions from an aqueous solution. Raw lignite was modified using *Fusarium lignite* B3, which successfully improved its physical and chemical properties, and its ability to adsorb heavy metal ions. This was attributed to increases in the pore size, specific surface area, and the number of active adsorption groups, such as carboxyl and phenolic hydroxyl groups. A heavy metal adsorption experiment proved that the BLA had good adsorption effects with Cu(II), Hg(II), Mn(II), and Cd(II), and the adsorption capacity was Cu(II) > Hg(II) > Mn(II) > Cd(II). The adsorption equilibrium time was 120 min and the maximum adsorption capacity for Cu(II) was 65 mg·g^−1^. The adsorption kinetics, SEM-EDS, and FTIR analysis demonstrated that chemisorption occurred between the BLA and heavy metal ions. The adsorption data were better fitted by the Freundlich model adsorption isotherm than the Langmuir isotherm model. The second-order kinetic models provided the best fit for the rate of Cu(II) adsorption onto the BLA. This work proposed an innovative method of fabricating low-cost adsorbents from lignite, and the microbiological method requires mild conditions that are relatively green compared to other techniques.

## Figures and Tables

**Table 1:** Basic characteristic of the lignite samples.

Chemical Properties	Experimental Results	
	Raw Lignite	BLA
C (%) ^a^	72.32 ± 1.36	64.63 ± 2.17
H (%) ^a^	4.87 ± 0.46	6.23 ± 0.81
O *(%) ^a^	20.43 ± 0.32	25.74 ± 1.02
N (%) ^a^	1.46 ± 0.15	1.97 ± 0.10
S (%) ^a^	0.92 ± 0.08	0.53 ± 0.11
M_ad_ (%)	11.63 ± 0.93	9.75 ± 0.71
A_ad_ (%)	15.41 ± 0.54	15.12 ± 1.19
V_ad_ (%)	38.77 ± 1.27	33.63 ± 0.61
FC *_ad_ (%)	34.19 ± 0.69	41.50 ± 1.26
BET (m^2^·g^−1^)	1.81 ± 0.32	5.66 ± 0.83

^a^ Water- and ash-free (900 °C). * FCad% = 100% − Mad% − Ad% − Vad%. * O% = 100% − C% − H% − N% − S%.

**Table 2:** Fitting parameters for pseudo-first-order and pseudo-second-order adsorption kinetics for Cu(II) with the BLA.

		Pseudo-First-Order			Pseudo-Second-Order		
Cu(II) (mg·L^−1^)	Qeexp (mg·g^−1^)	*k*_1_ (min^−1^)	*q*_e1_ (mg·g^−1^)	R_1_^2^	*k*_2_ (g·mg^−1^·min^−1^)	*q*_e2_ (mg·g^−1^)	R_2_^2^
50	11.48	0.107	11.3	0.950	0.181	11.5	0.991
150	27.5	0.037	28.2	0.932	0.032	27.5	0.976
300	56.1	0.033	56.4	0.927	0.013	56.0	0.977
500	64.5	0.042	61.7	0.981	0.0092	64.4	0.992
800	65.0	0.025	68.4	0.911	0.0097	66.3	0.982

**Table 3:** Langmuir and Freundlich parameters for Cu(II) metal sorption on the BLA at 24 °C.

	Langmuir Isotherm				Freundlich Isotherm		
Metal Ion	*Q_m_* (mg·g^−1^)	*K_L_* (L·mg^−1^)	*R_L_*	*R* ^2^	*K* (mg·g·L^1/n^·mg^1/n^)	*n*	*R* ^2^
Cu^(II)^	71.47	0.00649	0.10–0.88	0.95	3.52	2.31	0.91

**Table 4:** BET-specific surface area, cumulative pore volume, and average pore diameter of BLA with different particle sizes.

Sample	**Particle Size** Distribution (μm)	**BET Specific Surface Area (m^2^/g)**	Cumulative Pore Volume (cm^3^/g)	**Average Pore** **Diameter (nm)**
1	1000 > Φ > 825	7.66	0.06	24.08
2	825 > Φ > 425	6.35	0.05	23.70
3	425 > Φ > 250	6.12	0.04	22.61
4	225 > Φ > 150	5.76	0.04	19.64
5	150 > Φ	3.46	0.01	5.41

Note: the cumulative pore volume was calculated by the BJH method in the desorption process of 1.7–300 nm, and the average pore size was obtained by the BJH method in the N_2_ desorption process.

## Data Availability

Not applicable.
